# Psychophysical estimates guidelines for maximal acceptable loads during unilateral overhead work while wearing an exoskeleton in construction workers

**DOI:** 10.1371/journal.pone.0357036

**Published:** 2026-09-23

**Authors:** Maria Cristina Herrera Valerio, Mohammad Howard-Azzeh, Marcus Yung, Amin Yazdani

**Affiliations:** Canadian Institute for Safety, Wellness, and Performance, Cambridge, Ontario, Canada; PLOS, UNITED KINGDOM OF GREAT BRITAIN AND NORTHERN IRELAND

## Abstract

**Introduction:**

This study examined how passive shoulder exoskeletons, body archetype, sex, and shoulder elevation influence maximal drill-weight selection during overhead tasks.

**Methods:**

Twenty-nine construction workers (11 females, 18 males) completed high-frequency drilling every 12 seconds at a target force of 70 N across three shoulder elevations (90°, 120°, 150°), each performed for 5 minutes with and without a passive exoskeleton. Participant anthropometrics were captured using a 3D body scanner, and body archetypes were derived through k cluster analysis following variable extraction. A Psychophysical approach was used to determine maximal acceptable drill weight.

**Results:**

Mixed linear regression showed exoskeleton use increased selected weight by 336 g (~18%) (*p* < 0.001) compared to not wearing an exoskeleton. Smaller body archetypes selected 317 g (~17%) less than larger builds (*p* < 0.001), while medium builds showed no difference (p = 0.63). Males selected weights 300 g (~16%) heavier than females (p = 0.030). Shoulder elevation had no significant effect.

**Conclusions:**

These findings highlight key anthropometric and equipment related factors shaping overhead task demands.

## Introduction

Construction workers face a heightened risk of developing musculoskeletal disorders (MSD) compared to many other occupations. Shoulder injuries account for approximately 12% of all reported MSD cases in Ontario [1]. One of the primary contributors to work-related shoulder injuries is overhead work, defined as tasks involving shoulder flexion or abduction exceeding 60° [2]. These tasks place considerable strain at the shoulder level due to repetitive, sustained exertions performed in awkward postures [3]. Prolonged exposure can lead to a range of shoulder pathologies, including impingement syndrome, rotator cuff injuries, tendonitis, and localized muscle fatigue. Such conditions may result in temporary or permanent work disability [3]. Notably, evidence shows the risk of shoulder injury may double when the arm is elevated above 90° for more than 10% of a work shift, compared to tasks performed below shoulder level [4,5].

Common ergonomic risk assessment methods for assessing MSD risk at the upper extremity include self-reports, observational tools (e.g., rapid upper limb assessment [RULA], American Conference of Governmental Industrial Hygienists Threshold Limit Value Hand Activity Level [ACGIH TLV HAL]), direct measurements (e.g., electromyography [EMG], inertial motion units [IMU]), ergonomic software (e.g., 3D Static Strength Prediction Program) and biomechanical models [6]. While each method has strengths, they also face limitations such as subjectivity, lack of force sensitivity, complexity, or limited applicability to specific body regions [6]. Modeling the shoulder complex is particularly challenging due to its biomechanical complexity. Despite advancements, few approaches can dynamically and practically assess both posture and exerted force at the shoulder, highlighting the need for more feasible and comprehensive assessment strategies.

Given the substantial burden of shoulder injuries among construction workers, driven largely by overhead tasks that impose significant biomechanical strain, a thorough evaluation of the shoulder complex is essential. However, comprehensive assessments that integrate factors such as maximum acceptable loads, shoulder elevation angles, body archetypes, and assistive technologies remain scarce.

The Psychophysical approach offers a practical and widely used method for assessing work demands by integrating postural requirements and exerted forces through worker-perceived load tolerance. While psychophysics is inherently subjective and dependent on individual perception [7], it has been shown to provide reproducible and occupationally relevant estimates of maximum acceptable exertion levels when applied under controlled protocols [8]. As such, this approach has been used to determine maximally acceptable exertion loads for workers to minimize overexertion [9,10]. This approach closely mirrors real-world job tasks and helps determine safe exertion thresholds for manual material handling performed at low to moderate frequencies [11–13]. Psychophysics has been applied to determine the maximum acceptable weight of activities for several tasks such as lifting, lowering, carrying, pushing, and pulling [8].

There is limited research on the application of the Psychophysical approach to obtain upper extremity and/or shoulder complex safe weight limits. Previous research has examined the maximum acceptable weight for bilateral lifting at shoulder level, as well as from knuckle and floor levels [8]. Steele et al. [14] updated the pushing guidelines by shifting the focus towards the shoulder complex instead of the lower back, while Cudlip et al. [9] assessed downward exertion and pulling unilateral exertions at chest level. However, a significant gap remains in the literature regarding psycho-physically derived safety thresholds for unilateral overhead lifting, despite its relevance to many construction tasks.

Building on this gap, another under-explored factor is the influence of individual body archetypes and the use of assistive technologies, such as passive shoulder exoskeletons, on the maximum acceptable weights for unilateral overhead lifting. Exoskeletons have been shown to reduce muscle activity, enhance endurance, and improve task performance across various industrial applications [15,16]. Occupational exoskeletons developed for overhead tasks are generally categorized as either passive or active systems. Active exoskeletons rely on electric motors or pneumatic actuators to provide assistive torque; yet, these systems are often bulky, heavy, and limited in portability [17,18]. In contrast, passive exoskeletons generate assistive torque by storing potential energy in elastic elements, such as springs, and converting changes in arm elevation into mechanical assistance [18]. Passive shoulder exoskeletons commonly route arm-support forces through a posterior frame and hip interface; nonetheless, these systems are not designed to provide direct lumbar assistance.

Shoulder-support exoskeletons have been shown to reduce electrical activation of shoulder flexor muscles, such as the anterior deltoid, during sustained overhead work, suggesting potential reductions in fatigue and muscular demand [19]. However, the extent to which these physiological benefits translate into increased or safe maximal load-handling capacity remains unclear. Existing studies predominantly focus on muscle activation, metabolic cost, or kinematic adaptations under controlled loading conditions, but do not establish maximum acceptable or safe load limits for overhead tasks performed with exoskeleton assistance [20].

User acceptance remains a critical determinant of successful exoskeleton adoption. Field studies in construction work show that while workers often report reduced fatigue during sustained overhead tasks, usability factors such as fit, thermal discomfort, and interference with tool belts or harness may limit prolonged use without appropriate task matching and worker adaptation [21]. Despite the rapid growth in construction focused exoskeleton research, much of the existing evidence is derived from short duration, controlled evaluations that primarily emphasize biomechanical outcomes such as muscle activation, joint loading, and kinematics, while providing limited information on task-specific exposure limits relevant to real-world use.

Existing research has begun to bridge this exoskeleton guideline gap, particularly with respect to task-level parameters such as overhead work frequency. For example, Psychophysical evidence indicates that sustainable overhead work pace depends on both tool mass and exoskeleton design, with males selecting ~7–8 actions/min and females ~5–6 actions/min with a lighter tool [22]. Preliminary findings from a field study conducted by this team, examining work exposures and fatigue outcomes of construction workers wearing and not wearing a passive shoulder exoskeleton over two consecutive days, revealed that overhead activity was greater while using an exoskeleton [23]. Studies have also shown that tool mass can influence the effectiveness and impact of exoskeleton use [16,22,24,25]. However, while frequency and tool-related guidance is beginning to emerge, psycho-physically determined load thresholds for unilateral overhead work remain unclear. To avoid overexertion and promote safety standards for workers using exoskeletons, it is essential that clear and evidence-based guidelines be developed and implemented.

Prior research has demonstrated that body archetypes significantly impacted internal joint and skeletal loading during physical tasks [23]. Although some studies have examined the relationship between body archetypes and exoskeleton fit or user-device interaction [26], existing exoskeleton research has largely focused on biomechanical outcomes across different devices and task conditions. As a result, limited evidence is available on how individual body archetypes influence psycho-physically determined load tolerance during unilateral overhead lifting tasks, either with or without the use of passive exoskeletons.

Despite extensive use of Psychophysical methods to establish maximum acceptable loads for manual material handling tasks, existing research has largely focused on bilateral lifting performed below shoulder level. To date, few studies have applied a Psychophysical approach to unilateral overhead tasks, which differ fundamentally in biomechanical demand due to asymmetric loading and sustained shoulder elevation, and among the primary contributors to work-related shoulder injuries. These characteristics make unilateral overhead work particularly relevant to construction trades yet,remain poorly represented in existing Psychophysical guidelines.

The purpose of this study is to determine the estimated maximum acceptable loads in unilateral overhead lifting tasks using a Psychophysical approach. In addition, it seeks to investigate how these maximal loads are influenced by individual body archetypes and using a passive shoulder exoskeleton in both males and females in the skilled trades.

## Materials and methods

### 2.1 Study overview

The current study reports the estimated maximum acceptable loads for unilateral overhead lifting tasks from waist to above head level using a Psychophysical approach. Drilling was selected as the representative unilateral overhead task because it is widely performed across construction trades [21,27] and is strongly associated with elevated shoulder loading and injury risk [28]. Although the movement pattern in this study includes a drilling like component, the objective was not to evaluate drilling precision or task accuracy. Instead, drilling was chosen because it provides a realistic unilateral overhead posture in which load manipulation is central to the physical demand of the task.

Participants met with researchers across three separate sessions, spaced at least 24 hours apart (i.e., familiarization, intervention day 1, and intervention day 2) to minimize residual fatigue effects between conditions. The study employed a mixed-effect design with repeated measures and involved simulated fixed-frequency drilling tasks performed at shoulder elevations of 90°, 120°, and 150°. Each condition was tested under both control (no exoskeleton) and passive exoskeleton interventions. The starting drill weight was either 1 kg (representing battery weight) or 3 kg. Participants self-selected the drill weight before and at regular intervals during each condition. To control for order effects and potential confounding variables, the intervention type, shoulder elevation condition, and drill weight were randomized.

### 2.2 Participants

Females and males with experience in any construction trade or sector were recruited to participate in this study. Individuals were excluded if they were under 18, or had severe musculoskeletal issues in the shoulders, arms, back, or hands; skin diseases, injuries, sensitivities to patches, or raised scars with swellings; or sensory and circulatory disorders in the upper extremities, hips, or back (e.g., diabetic neuropathy or lymphatic drainage disorders).

To estimate the adequate sample size to compare the difference in estimated maximum acceptable load between exoskeleton and control interventions within the same participants. The a priori sample size was determined using a power calculation for a paired (within-subject) comparison of means, based on the expected mean within-person difference and the standard deviation of the within-person differences, using α = 0.05 and 80% power. Results of the calculation indicated that 29 participants would be required. In this study, a total of 29 construction workers (11 females, 18 males) from 10 different trades participated. No participants in this study identified as a gender other than male or female. This study was approved by the Conestoga College Ethics Board (REB 541), and written informed consent was obtained from all participants prior to participation. Recruitment period started on August 1, 2024 and finalized on March 1, 2025.

### 2.3 Experimental procedures

#### 2.2.1 Familiarization and 3D anthropometric assessment.

Potential participants were screened for eligibility prior to the start of the study. Those who met the criteria completed a demographics questionnaire, underwent an anthropometric full body scan, and participated in a familiarization session involving orientation on using the exoskeleton and practicing the experimental task. This familiarization session lasted approximately 20–30 minutes and was designed to ensure participants were comfortable with the exoskeleton, familiar with the tasks, and demand familiar with the experimental procedures prior to intervention days.

During the familiarization session, participants received a standardized explanation of the study protocol, including the purpose of the exoskeleton, the overhead drilling task, and the sequence of experimental conditions. Participants were then fitted with the upper extremity exoskeleton (Hilti EXO-02; Hilti, Schaan, Liechtenstein) following the recommendations outlined in Du et al. [21]. To ensure a proper fit, researchers confirmed that:

- Waist straps were snugly secured around the iliac crest.
- Ball sockets and articulation units were aligned with the contour of each participant’s torso.
- The upper edge of the articulation unit was positioned at shoulder height.
- The back plate was centered between the shoulder blades.
- The tension cord formed a wide V-shape across the upper back.

Arm support levels were adjusted based on participant preference, with instructions to select a resistance setting that provided adequate support while still allowing natural arm movement without excessive force.

Participants performed several practice trials of unilateral overhead drilling task at each shoulder elevation condition using a drill, with and without an exoskeleton.

Shoulder elevations at 90°, 120°, and 150° were measured using a joint angle-measuring goniometer, along with an assessment of participant preferred foot position for drilling at each angle. Participants selected a foot placement where they felt most comfortable (marked with tape) and were instructed to remain in that location throughout the data collection. When determining foot placement, participants were instructed to select a preferred foot position and use natural movement strategies to enhance task realism and to reflect how overhead drilling is typically performed in occupational settings. Foot position was selected during the familiarization session, marked, and held constant across all experimental conditions for each participant, ensuring that postural variation occurred primarily between participants rather than within participants. For each shoulder elevation condition, the distance from the taped position to the frame was recorded.

Afterwards, an anthropometric body scan was performed (MOVE 4D, IBV, Valencia, Spain). Previously, this 3D scan has demonstrated accuracy when measuring body dimensions [29–31]; for this study, the body scans were used to characterize individual body shape and segment proportions relevant to shoulder mechanics and exoskeleton fit, supporting analyses of body archetype–related differences in neuromuscular response during overhead work. Participants were asked to assume an ‘A’ pose (feet spread apart, arms relaxed and angled slightly outward from the body) and to remain still for 10 seconds during a scan. The scanner does not require participants to wear markers or to disrobe. The outputs of the scanning were discrete anthropometric measurements of all body segments.

#### 2.2.2 Intervention Days 1 and 2.

**Fig 1:**
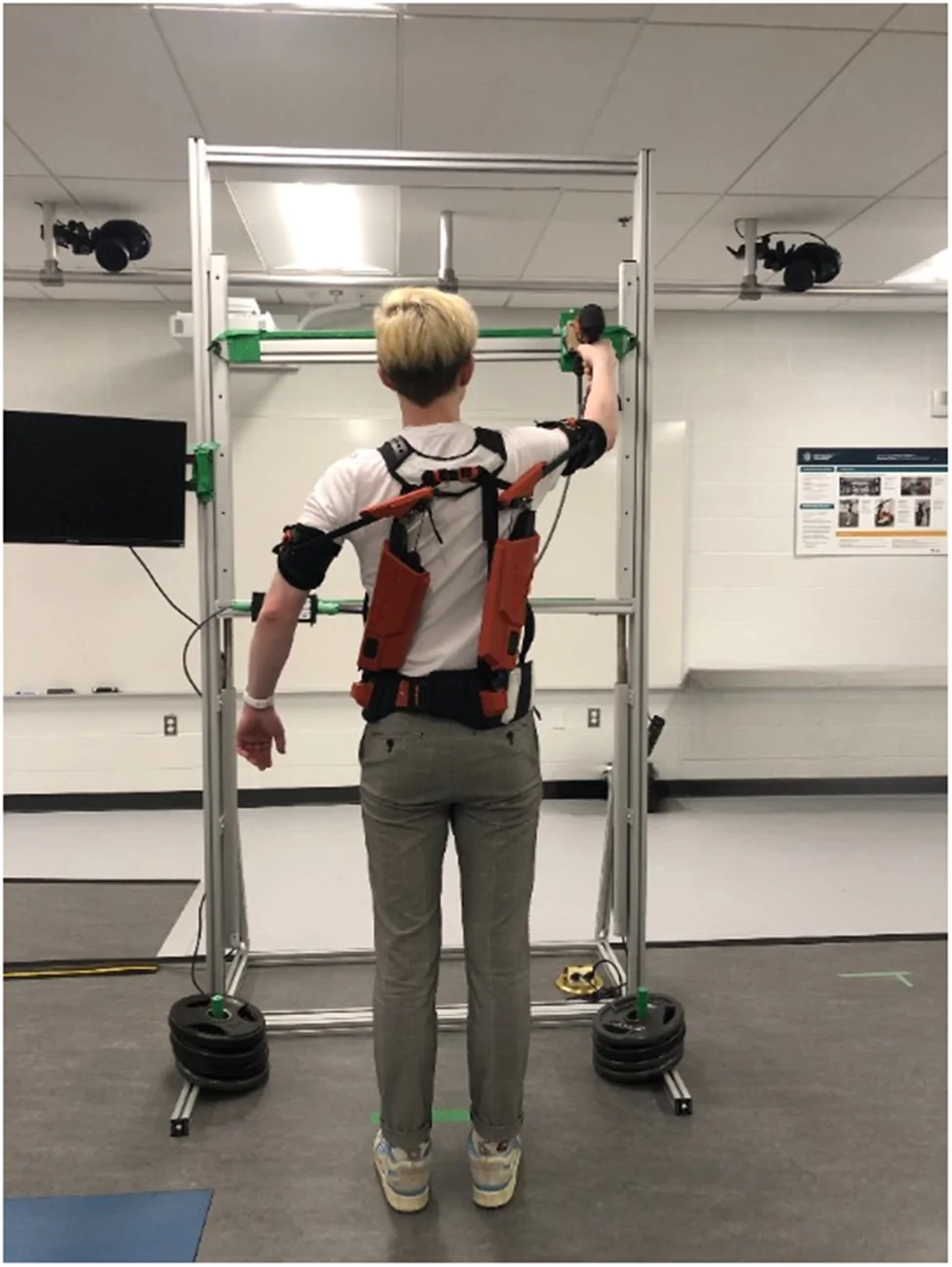
Participant performing a simulated drilling task under the exoskeleton intervention. Alt text: A participant performing a simulated overhead drilling task while wearing an upper-body passive shoulder exoskeleton for physical support, in a laboratory environment designed for ergonomic evaluation.

**Fig 2:**
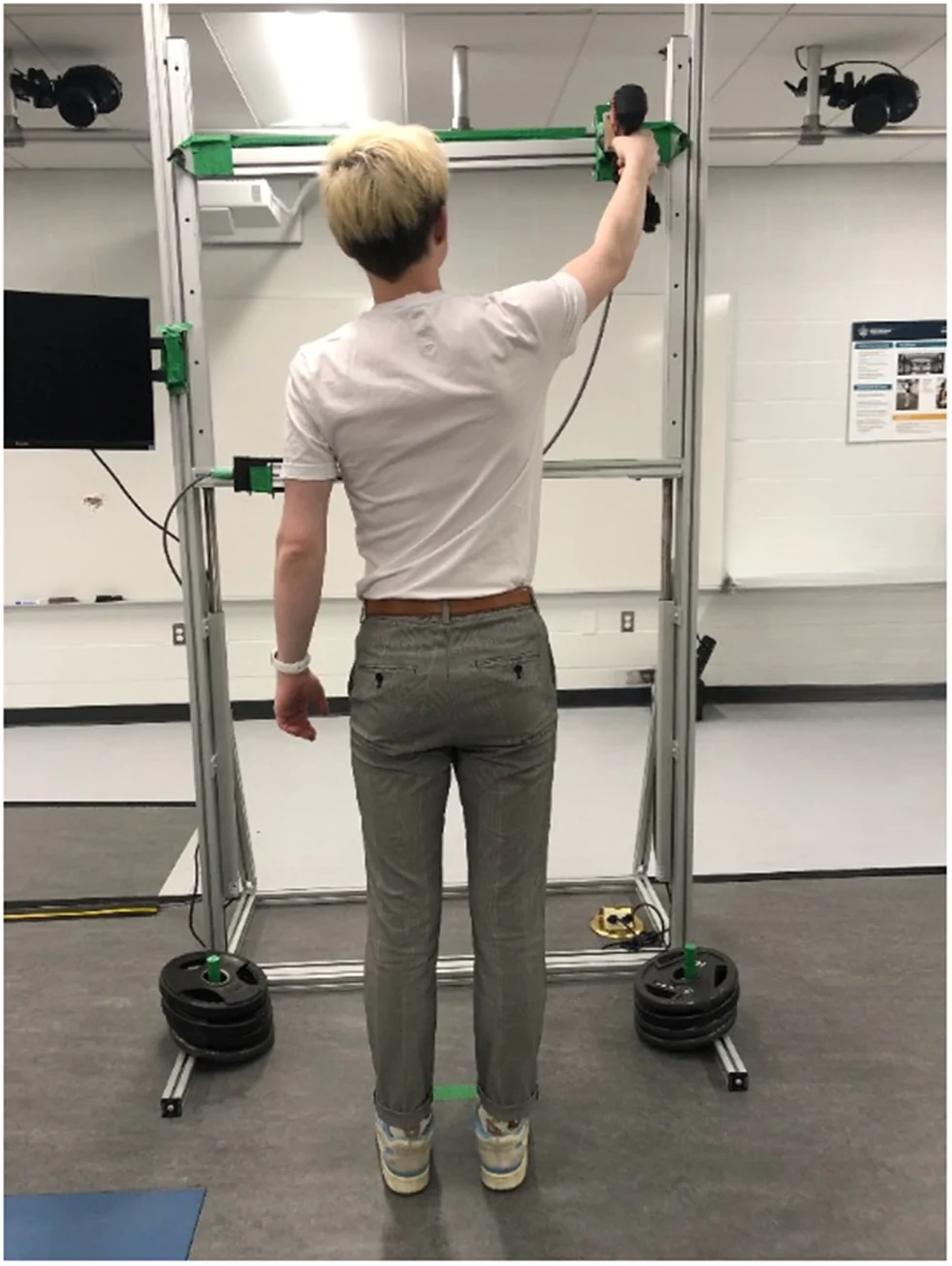
Participant performing a simulated drilling task under the control intervention (no exoskeleton). Alt text: A participant performing a simulated overhead drilling task without assistive equipment under control conditions, in a laboratory environment designed for ergonomic evaluation.

Simulated drilling tasks were performed using a custom-built frame with adjustable height across two separate interventions, one with and one without the exoskeleton (Days 1 and 2) (Fig 1, Fig 2). The individuals pictured in Fig 1 and Fig 2 have provided written informed consent (as outlined in PLOS consent form) to publish their images alongside the manuscript. The two-day experimental structure was implemented to reduce the influence of transient fatigue. The drilling target was mounted to a load cell sampled at 1000 Hz (Bertec 4060, Columbus, USA) and a nearby monitor displayed real-time force data as a graphical output.

Participants were instructed to drill the target upon hearing an audible beep, aiming for 70 N of force, representing a realistic load typically applied during manual drilling operations [32,33]. The monitor provided immediate visual feedback during each trial. Once participants reached the target force, the researcher signaled audibly for the participant to maintain the drilling force for 2 seconds before returning to the initial position. If participants did not reach 70 N, an audible cue was provided at the maximum exerted force recorded by the load cell.

The audible beep was programmed to occur every 12 seconds, creating a high-frequency work cycle consisting of 7 seconds of active engagement followed by 5 seconds of rest. Drilling tasks were carried out under three shoulder elevation conditions: 90°, 120°, and 150°, with each condition lasting 5 minutes, with a break of 2 minutes between condition. The task duration of 5 minutes and the 12-second drilling intervals were informed by a previous study that examined typical patterns of overhead drilling, including daily frequency and cumulative exposure times [27]. Following established Psychophysical methodologies [7,34], participants selected loads during short duration conditions based on their perceived ability to sustain the task over an 8hour work shift. Thus, estimated maximum acceptable loads reflect perceptual tolerance rather than prolonged physiological capacity. The sequence of interventions and experimental conditions was randomly assigned for each participant to minimize order effects and potential biases.

The experimental drill was a 3D-printed replica of a real battery powered hand drill. The drill had a fixed unloaded weight of 1 kg to represent the minimum tool weight, and its total weight was adjusted by adding or removing lead pellets into the hollowed core of the drill. To eliminate visual feedback on drill weight, participants were blind to the amount of lead shot within the drill.

For each session, participants selected their own drill weight following instructions from the researcher. They were asked to imagine performing drilling tasks over an 8-hour shift and to work as efficiently as possible without experiencing undue strain. Based on sensations in their shoulder, arms, back, and neck, participants could adjust the load by adding or removing lead pellets. Participants were allowed to modify the weight between trials and during a trial if needed. If a participant wished to adjust the weight during an ongoing trial, the experiment was briefly paused to allow the adjustment, after which the trial resumed at the same prescribed frequency. Load adjustments were encouraged both before and throughout each experimental condition to help participants find an optimal balance that allowed sustained work without fatigue or discomfort. At least once during each condition, participants were explicitly asked whether they wanted to adjust the weight.

Following established Psychophysical adjustment methodologies, participants were permitted to adjust the drill weight as often as desired both prior to initiating each condition and during task execution. Adjustments were based on participants ability to sustain the prescribed task demands without undue discomfort or fatigue over an imagined 8-hour work shift. No fixed limit was imposed on the number of adjustments, allowing participating to refine the load selection until an acceptable balance was achieved.

For each shoulder elevation condition, the final selected weight was defined as the drill weight at the end of the 5-minute condition, provided that the participant did not request further adjustment during the final minute of in use the task. This performance load value was taken to represent the participants estimated maximum acceptable load for that condition. If additional adjustments were made earlier in the condition, only the final value was retained for analysis.

**Fig 3:**
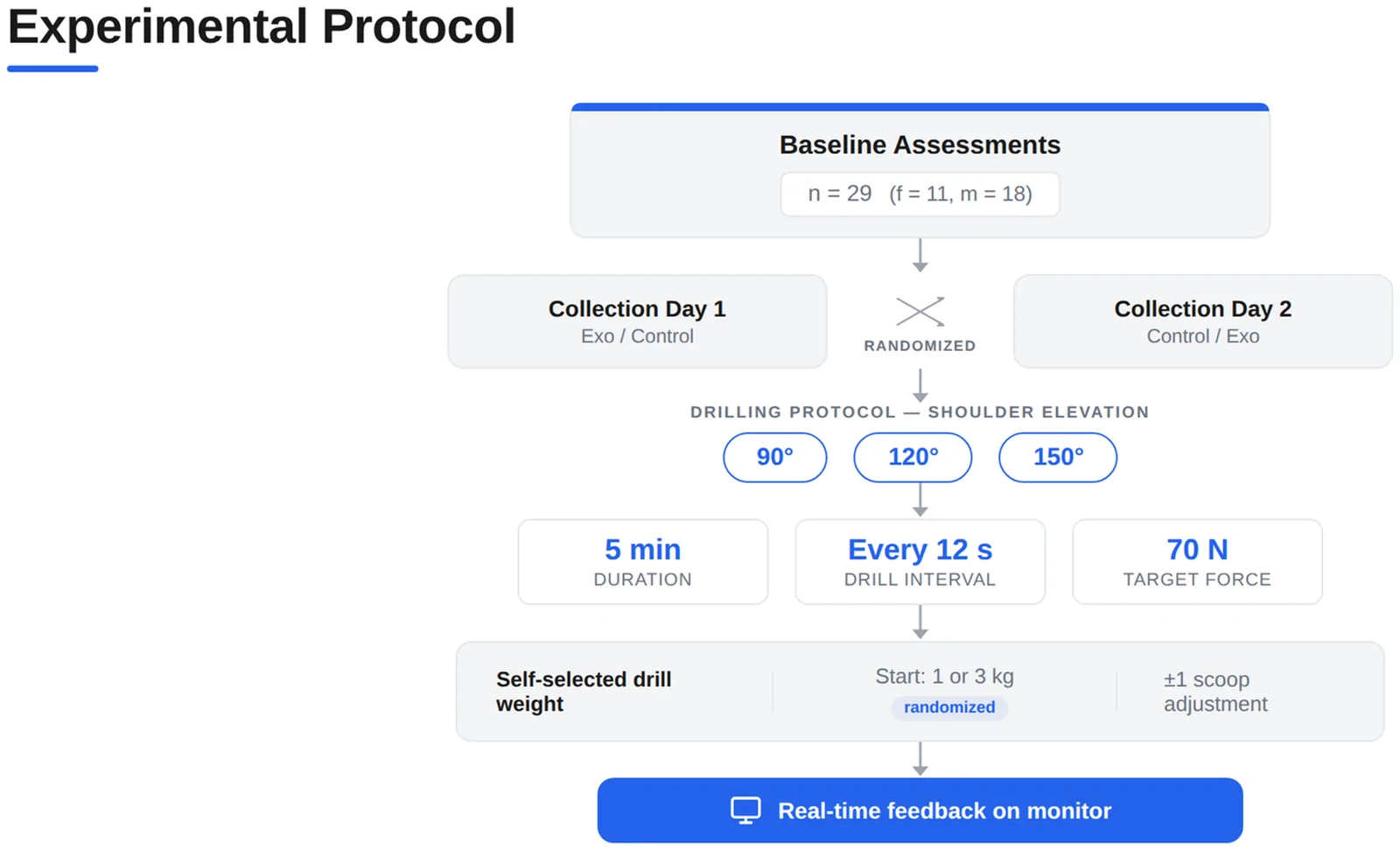
Workflow of psychophysical study simulating drilling tasks at 90°, 120°, and 150° shoulder elevation conditions in both control and exoskeleton interventions. Alt text: Flowchart illustrating the workflow of a Psychophysical study involving simulated drilling tasks at 90°, 120°, and 150° shoulder elevation. The protocol includes baseline assessments, randomized weight conditions, and task execution under both control and exoskeleton interventions with construction worker participants.

To reduce potential starting-point bias, expectation, and carryover effects between experimental conditions [35], a randomized load offset (~350 g) was introduced prior to each condition. Specifically, researchers avoided predictability in their interactions by randomly adding or removing scoops of lead shot (~350 g) between conditions.small This adjustment was intended to encourage independent load selection across conditions while remaining sufficiently small to avoid constraining or materially influencing participant subsequent weight adjustments during the task. The initial drill weight was randomized to either 1 kg or 3 kg. The rationale for selecting two initial drill weights follows established Psychophysical methodology in which participants begin from either a low load or high load condition to minimize starting-point bias effects and facilitate adjustment toward an acceptable workload [36]. While prior Psychophysical studies have defined these starting points using population based percentiles (e.g., 10th and 90th percentiles from the Snook Tables) [36], the present study employed task-specific, equipment based starting load conditions. Specifically, the 1 kg condition represented the minimum realistic mass of a battery powered drill, whereas the 3 kg condition slightly exceeded the mass of a typical cordless drill, thereby approximating an upper realistic boundary for overhead drilling tasks. To ensure methodological consistency, a randomized assignment of weight conditions across intervention days was implemented. Each participant was randomly assigned either 1 kg or 3 kg on Intervention Day 1. On Day 2, participants underwent a fresh randomization, meaning they could receive the same weight condition as Day 1 or a different one. Additionally, half of the participants began with the 1 kg condition and half with the 3 kg condition on Day 1. This counterbalancing helped control for order effects. The workflow of the experiment is illustrated below (Fig 3).

### 2.3 Data processing

#### 2.3.1 Anthropometric measurements.

Thirty-six anthropometric measurements spanning all body segments were extracted from each 3D scan. To explore body shape archetypes relevant to exoskeleton design, a clustering analysis was conducted using five selected measures: height, waist girth, upper arm length, upper arm girth, and bust girth. These variables were selected because they capture key dimensions known to influence exoskeleton interface alignment, load transfer, and fit at the torso and upper extremity. To improve robustness and reproducibility of the clustering analysis, several procedural controls were implemented. Highly correlated anthropometric variables were identified through correlation analysis and excluded to reduce dimensional redundancy. All variables were standardized prior to clustering, and analyses were conducted separately for male and female participants to avoid sex related scaling effects. Candidate cluster solutions were evaluated using the Elbow Method based on within cluster sum of squares. The k means algorithm was run with multiple random initialization to reduce sensitivity to starting conditions, and final cluster solutions were selected based on convergence behavior, visual separation, and interpretability of anthropometric profiles. This approach to obtain body archetypes based on exoskeleton fit was created based on Riemer [26].

The analysis employed the k-means clustering algorithm (R Studio 2024.12.0), with the number of clusters determined separately for male and female participants using the Elbow Method [35], based on the within-cluster sum of squares (WSS). Three clusters were identified for each sex. Participants were treated as data points within a multi-dimensional space defined by the selected measures. Initial cluster centroid were randomly assigned, and participants were iteratively reclassified to the nearest centroid as the cluster centers updated. This process continued until the cluster assignments stabilized and no longer changed.

Several cluster configurations were tested, and the final model was selected based on optimal visual separation and interpretability. Upon final clustering, the mean and range values for each cluster were calculated to characterize distinct body shape profiles. After defining the clusters and their ranges, each participant individual measurements were analyzed and assigned to the appropriate cluster. Final assignment was based on mode (i.e., the cluster that repeated the most from all five selected measures). Clusters (i.e., archetypes) were categorized as larger build, medium build, or smaller build categories.

#### 2.3.2 Estimated maximum permissive unilateral overhead loads.

Estimated maximum permissive overhead loads were organized by sex (i.e., male, female) and intervention type (i.e., control, exoskeleton). Load values were specified for shoulder flexion angles of 90°, 120°, and 150° to represent varying overhead working positions.

The initial step involved determining estimated maximum permissive loads across conditions. The dataset was stratified into percentile groups (i.e., 10th, 25th, 50th, 75th, 90th) using a threshold derived from the sample. Using R Studio (version 2024.12.0), metrics were computed based on intervention, condition, and sex, including the drill weight threshold at the specified percentile. These calculations were performed separately for each sex under both experimental interventions. Consistent with prior ergonomics design guideline tables [8], the reported percentiles are intended to support relative comparisons across conditions [37] rather than to define externally validated injury thresholds. The reported load estimates were derived from a limited sample under controlled, short-duration laboratory conditions and should be interpreted as preliminary, task-specific benchmarks rather than generalized field limits.

### 2.4 Statistical analysis

To examine the associations between predictor (dependent) variables, including sex, intervention group (i.e., with exoskeleton vs. without exoskeleton/control), shoulder elevation condition (i.e., 90°, 120°, and 150°), body archetype from 3D scanner (i.e., larger, medium, and smaller build), foot to frame distance, and age, with drill weight selection, the data were fitted into a multivariable mixed linear regression model as follows. Correlation between independent variables was assessed using appropriate correlation coefficients (i.e., Pearson, Phi, or Spearman rank) based on variable type. If two variables were correlated above |0.75|, the more epidemiologically plausible variable was retained for further modeling; however, no correlations exceeded this threshold. Linearity between continuous predictors and selected drill weight was examined graphically using locally weighted scatterplot smoothing (LOWESS) curves. Nonlinear predictors would have been categorized, but age (the only continuous variable) demonstrated a linear relationship with the outcome.

Univariable mixed linear regression models with random intercepts for each participant were fitted to account for the repeated-measures design of this study. Independent variables associated with drill weight at α = 0.05 were considered for inclusion in the multivariable model. Forward selection was then applied, adding predictors sequentially from most to least significant based on univariable results, while also considering epidemiological plausibility. For variables with more than two categories, overall significance was assessed using Wald’s $\chi^{2}$ test. Variables that did not meet the statistical criteria during forward selection were reintroduced into the model to assess confounding. If the inclusion of a variable resulted in a ≥10% change in the coefficient of another significant variable, and the relationship was consistent with causal criteria, it was retained as a con-founder (i.e., if the effect was reduced) or as a distortion variable (i.e., if the effect increased or changed direction). Predictors were retained if statistically significant (α = 0.05) or demonstrated confounding or distortion effects on other variables.

The final multivariable linear regression model with random intercepts for participant ID to account for repeated measurements was used to examine the associations between the independent variables and selected drill weight. The model was expressed as:


$$\begin{array}{ccc} Y_{ij} & = & \beta_{0}+\beta_{1}({\text{Exoskeleton}}_{ij})+\beta_{2}({\text{MediumBuild}}_{ij})+\beta_{3}({\text{SmallBuild}}_{ij})\\ & & +\beta_{4}({\text{Male}}_{ij})+\beta_{5}({\text{Condition\#120}}_{ij})+\beta_{6}({\text{Condition\#150}}_{ij})\\ & & +\beta_{7}({\text{Age3}}_{ij})+u_{j}+e_{ij} \end{array}$$

where *i* indexes observations and *j* indexes participants; $Y_{ij}$ represents the predicted drill weight for observation *i* from participant *j*; $u_{j}$ represents the participant-level random intercept, and $\epsilon_{ij}$ represents the residual error.

Model diagnostics included assessment of outliers using Pearson and deviance residuals. Assumptions of normality and homoscedasticity of random intercepts were evaluated using best linear unbiased predictions (BLUPs) at the individual level, and residuals were also examined for normality and homoscedasticity. The analysis was performed in Stata 19 (StataCorp, College Station, TX).

## Results

Participant demographics and descriptive statistics of the sample are included in Table 1.

**Table 1:** Descriptive statistics and demographic information.

	Males (n = 18)	Females (n = 11)
Age (years)		
Mean (SD)	23 (5)	28 (5)
Median [IQR]	22 [20; 24]	29 [23; 32]
Body mass (kg)		
Mean (SD)	80.38 (16.87)	79.22 (17.45)
Median [IQR]	78.93 [66.68; 86.18]	72.57 [68.04; 88.45]
Height (cm)		
Mean (SD)	173.7 (7.1)	168.5 (6.3)
Median [IQR]	175.5 [170.5; 178.4]	166.9 [163.6; 175.3]
Trade		
Carpentry	4	6
Concrete finisher	1	0
Electrician	7	1
General labor	0	1
General machinist	1	0
HRAC	0	1
Instrumentation and Control Technician	0	1
Millwright	2	0
Plumbing	2	1
Welding	1	0
Level (Apprentice = Individual registered in a formal training program combining technical instruction and supervised workplace experience. Labour = individual engaged in general labour with no certified training. Journeyperson = certified tradesperson who has completed an apprenticeship program and passed the required certification exams.)		
Apprentice	9	7
Worker	6	4
Journeyperson	3	0
Years of Experience		
Mean (SD)	3 (3)	4 (6)
Median [IQR]	2 [3]	2 [4]

Diagnostic evaluations of the model indicated that the assumptions of linearity, homoscedasticity, and normality were reasonably satisfied, supporting the validity of the linear mixed modelling approach.

Table 2 presents

**Table 2:** Summary statistics. Mean (±) SD; Median [IQR] and archetypes for key anthropometric measures by sex.

Measure	Mean SD (cm)	Median IQR (cm)	Archetypes		
			Cluster 1- Larger build (cm)	Cluster 2- Medium build (cm)	Cluster 3- Smaller build (cm)
FEMALES					
Height	168.5 (6.2)	166.9 [163.6; 175.3]	160.8 - 167.9	159.9 - 172.3	174.6 - 179.1
Bust girth	107.2 (13.4)	103.9 [100.3; 113.7]	113.8 - 135.2	82.6 - 104	94.6 - 109.1
Upper arm length left	28.6 (1.4)	28.4 [27.4; 29.9]	27.1 - 28.6	26.5 - 28.7	29.7 - 31.2
Upper arm length right	28.5 (1.5)	28.4 [27.4; 29.9]			
Upper arm girth left	32.7 (4.3)	32.5 [29.4; 37.0]	35.8 - 40.8	25.6 - 31.7	26.1 - 33.2
Upper arm girth right	32.4 (4.7)	31.7 [28.7; 35.8]			
Waist girth	91.1 (1.6)	86.2 [77.8; 101.4]	101.4 - 120.8	61.8 - 94.1	73 - 92.2
MALES					
Height	173.7 (7.1)	175.5 [170.5; 178.5]	172.4 - 187.3	170.5 - 181.8	158.6 - 178.5
Bust girth	101.2 (15.0)	96.3 [90.8; 111.4]	90.8 - 103.6	114.3 - 138.4	81.7 - 111.4
Upper arm length left	31.2 (1.7)	31.8 [30.0; 32.4]	31.6 - 33.6	31.6 - 33.2	28.6 - 30.5
Upper arm length right	31.2 (1.6)	31.7 [30.0; 32.2]			
Upper arm girth left	32.9 (5.4)	31.8 [29.1; 35.0]	27.6 - 34.3	36.7 - 45	24.9 - 35.4
Upper arm girth right	32.8 (5.0)	32.1 [28.8; 34.5]			

Estimated maximum permissive overhead loads

Across sexes, shoulder elevation angles, and body archetypes, percentile-based results in Table 3 show a consistent increase in estimated maximum permissive drill weight with exoskeleton use compared to the controls. This pattern indicates that overall, exoskeleton assistance increased the acceptable load while preventing undue discomfort and fatigue. Additionally, larger body archetypes consistently tolerated higher loads than medium or smaller builds, while males demonstrated higher acceptable loads than females. There were small differences across shoulder elevation angles, suggesting that this variable had a minor influence on maximal permissive load. These descriptive trends were formally evaluated using a multivariable linear mixed-effects regression model (Table 4) to quantify the independent contributions of intervention type, body archetype, sex, age, and shoulder elevation angle.

**Table 3:** Estimated psychophysically-determined maximum permissive unilateral overhead loads (kg) by condition, sex, shoulder elevation, and drill weight percentile.

**A. Control Males**										
Control Males Maximum Drill Weight (kg)										
Shoulder elevation (°)	90	120	150	Percentile (%)						
	1.40	1.31	1.40	10						
	1.53	1.60	1.50	25						
	2.00	1.90	2.00	50						
	2.35	2.53	2.37	75						
	2.87	2.76	2.62	90						
Control Males Archetype-specific Maximum Drill Weight (kg)										
Shoulder elevation (°)		90			120			150		Percentile (%)
Archetype	1	2	3	1	2	3	1	2	3	
	1.98	1.4	1.43	2.23	1.34	1.36	1.99	1.44	1.37	10
	2.05	1.57	1.50	2.34	1.55	1.50	2.12	1.53	1.40	25
	2.16	2.00	1.83	2.54	1.90	1.60	2.35	2.00	1.75	50
	2.25	2.33	2.29	2.71	2.53	2.40	2.65	2.36	2.24	75
	2.32	2.63	2.68	2.82	2.66	2.78	2.83	2.54	2.67	90
**B. Exoskeleton Males**										
Exoskeleton Males Maximum Drill Weight (kg)										
Shoulder elevation (°)	90	120	150	Percentile (%)						
	1.78	1.65	1.57	10						
	1.95	2.10	1.98	25						
	2.46	2.73	2.37	50						
	2.74	2.98	2.83	75						
	2.89	3.00	3.00	90						
Exoskeleton Males Archetype-specific Maximum Drill Weight (kg)										
Shoulder elevation (°)		90			120			150		Percentile (%)
Archetype	1	2	3	1	2	3	1	2	3	
	1.98	1.76	1.60	2.23	1.63	1.60	1.99	1.55	1.42	10
	2.05	1.93	1.76	2.34	1.99	1.90	2.13	1.83	1.55	25
	2.16	2.27	2.20	2.52	2.58	2.50	2.35	2.35	2.20	50
	2.25	2.76	2.58	2.71	2.92	2.81	2.65	2.66	2.54	75
	2.32	2.92	2.64	2.82	3.00	2.98	2.83	2.97	2.88	90
**C. Control Females**										
Control Females Maximum Drill Weight (kg)										
Shoulder elevation (°)	90	120	150	Percentile (%)						
	1.20	1.30	1.30	10						
	1.25	1.60	1.50	25						
	1.60	1.70	1.80	50						
	2.22	1.95	1.85	75						
	2.40	2.10	2.00	90						
Control Females Archetype-specific Maximum Drill Weight (kg)										
Shoulder elevation (°)		90			120			150		Percentile (%)
Archetype	1	2	3	1	2	3	1	2	3	
	1.36	1.49	1.13	1.36	1.73	1.32	1.40	1.52	1.25	10
	1.45	1.92	1.17	1.45	1.77	1.50	1.55	1.70	1.47	25
	1.60	2.22	1.40	1.60	1.95	1.70	1.80	1.80	1.70	50
	1.87	2.33	1.80	1.62	2.13	1.87	1.85	1.85	1.88	75
	2.03	2.37	2.16	1.62	2.17	2.01	1.88	1.94	2.01	90
**D. Exoskeleton Females**										
Exoskeleton Females Maximum Drill Weight (kg)										
Shoulder elevation (°)	90	120	150	Percentile (%)						
	1.37	1.40	1.30	10						
	1.60	1.50	1.54	25						
	2.07	1.78	2.00	50						
	2.25	2.34	2.51	75						
	2.70	3.00	2.7	90						
Exoskeleton Females Archetype-specific Maximum Drill Weight (kg)										
Shoulder elevation (°)		90			120			150		Percentile (%)
Archetype	1	2	3	1	2	3	1	2	3	
	1.85	1.44	1.32	1.85	1.40	1.32	1.74	1.38	1.36	10
	1.94	1.54	1.50	2.08	1.40	1.50	1.84	1.51	1.45	25
	2.08	1.85	1.90	2.47	1.59	1.80	2.00	1.94	2.05	50
	2.54	2.25	2.22	2.73	2.09	2.07	2.35	2.33	2.62	75
	2.82	2.52	2.27	2.89	2.63	2.21	2.56	2.38	2.67	90

**Table 4:** Multivariable linear mixed regression model examining the associations between intervention (exoskeleton vs. control), body archetypes, sex, shoulder elevation, age, and body mass on the maximum permissive overhead load.

Measure	Coefficient	Std error	p value	CI 2.5%	CI 97.5%
Control	(Reference)				
Exoskeleton	0.34	0.06	<0.001*	0.21	0.45
Large build archetype	(Reference)				
Medium build archetype	−0.07	0.15	0.634	−0.38	0.23
Smaller build archetype	−0.31	0.13	0.023*	−0.59	−0.04
Females	(Reference)				
Males	0.29	0.07	0.030*	0.29	0.56
90° condition	(Reference)				
120° condition	0.03	0.07	0.653	−0.11	0.17
150° condition	−0.01	0.01	0.844	−0.15	0.12
Age	−0.01	0.33	0.238	−0.03	< 0.01

A multivariable mixed linear regression model was used to examine the effects of intervention, body archetype, age, sex, and shoulder elevation condition on drill weight (kg) (Table 4). The exoskeleton condition was associated with a statistically significant higher drill weight, an increase of 336 g compared to the control (i.e., without exoskeleton) (*p* < 0.001). Participants with a smaller body archetype selected a significantly lower drill weight (317 g less) compared to those with a larger build (*p* < 0.001), while the medium build archetype showed no significant difference when compared to smaller builds (p = 0.63). Male participants managed a significantly higher drill weight than females, with an average increase of 300 g (p = 0.030). There was no evidence to suggest shoulder elevation conditions at 120° and 150° were associated with more or less drill weight than at 90° (120°: p = 0.653; 150°: p = 0.844).

## Discussion

The current study identifies the estimated maximum acceptable loads for unilateral overhead lifting tasks using a Psychophysical approach. It is novel in its exploration of the effects of force-augmenting passive exoskeleton and anthropometric characteristics on load tolerance.

Overall, both male and female participants were able to lift heavier loads when using the exoskeleton, with an average increase of 340 g after controlling for several key variables, indicating a positive effect of the intervention. No significant differences in drill weight were observed across shoulder elevation angles of 90°, 120°, and 150°, suggesting that perceived exertion may not increase proportionally with elevation angle. Male participants selected significantly higher loads than females, with an average difference of 300 g. Similarly, individuals with a larger body archetype lifted 317g more than those with a smaller build. However, no significant differences were observed between medium and large builds.

For most participants (i.e., 90th percentile), the estimated maximum acceptable load for unilateral overhead tasks remained below 3 kg across all conditions, regardless of intervention. This threshold is only slightly above the weight of a standard drill, revealing a possible mismatch between safe lifting limits and the heavier loads and hand tool weights commonly used in construction.

These findings underscore the need for ergonomic interventions that extend beyond exoskeleton support. They also highlight the potential of exoskeletons to enhance unilateral overhead lifting capacity and perceived safety, particularly at higher shoulder elevations and among individuals, particularly males, with specific anthropometric characteristics. For practitioners and construction businesses, these results offer ergonomic guidance on overhead work, inform drill design, and support evidence-informed recommendations for exoskeleton use.

### 4.1 Estimated maximum permissive loads in unilateral overhead tasks

Across all conditions and interventions, the 90th percentile estimated maximum acceptable load remained below 3 kg. This is only marginally above the weight of a standard drill (i.e., about 2.4 kg). However, many workers in trades involving overhead work routinely handle heavier tools. For example, wire-coil drills commonly used in electrical work can weigh approximately 4.5 kg, and pipe insulation tasks often involve managing tools and materials exceeding 3 kg. Moreover, the weight continues to increase as manufacturers integrate advanced features. Cordless power tools, now predominantly powered by lithium-ion batteries, offer high energy density but add significant weight compared to older battery technologies [38]. Moreover, while the current experiment setup involved one arm overhead, real-world often tasks require use of the non-dominant hand to hold or manipulate additional components, further increasing physical demands.

To contextualize the observed load estimates related to existing ergonomic guidelines, it is useful to consider how manual material handling criteria differ from the present task. Widely used guidelines such as the National Institute for Occupational Safety and Health (NIOSH) Lifting Equation recommend weight limits based on posture, reach, frequency, and lift duration [39]. However, the NIOSH Lifting Equation was developed for two-handed, symmetric lifting tasks and does not explicitly account for unilateral loading and sustained overhead postures, and therefore, may not be directly applicable to unilateral overhead drilling tasks.The current study findings, which reflect substantially lower load capacities, highlight the importance of task-specific Psychophysical assessments when evaluating unilateral overhead work demands, not captured by traditional lifting models. Previous research comparing maximal acceptable weights derived from Psychophysical methods to those recommended by the NIOSH lifting equation has shown that the latter tends to be more conservative [40]. Therefore, while reference guidelines offer a valuable baseline, they should be applied with caution, and more task-specific ergonomic assessment tools are recommended when designing criteria for maximal load handling in overhead work.

### 4.2 Impact of exoskeleton use on perceived load tolerance

Both male and female participants lifted heavier loads when using the exoskeleton, with an average increase of 340 g across all conditions, indicating an increase in perceived load tolerance under the tested conditions. It should be noted that this increase reflects short-term task performance and not direct evidence of improved safety or injury risk reduction.

Prior research provides supporting context for these findings. Lower limb exoskeletons have been shown to augment human load carrying capacity by reducing biomechanical and physiological demands during loaded locomotion, thereby enabling users to sustain heavier effective loads or longer task durations than would otherwise be possible [41]. Additionally, recent field research has reported that workers wearing an upper body exoskeleton voluntarily chose to perform a greater amount of overhead work compared to when unassisted, suggesting that exoskeleton use can influence task selection in real work environments [42]. However, the direct examination whether users increase their self-selected external load when wearing an exoskeleton, remains limited. The present study addresses this critical gap by explicitly examining how exoskeleton use affects load selection behavior.

Literature supports these findings in related contexts, showing that shoulder exoskeletons can reduce muscular load, promote ergonomic posture, and improve endurance [43,44]. Notably, they have been shown to reduce shoulder discomfort by up to 57% and perceived exertion by 21.5% [15].

However, increased lifting capacity does not imply immunity to fatigue or overexertion. The exoskeleton weight and force may increase effort in non-targeted muscles such as the pelvis and lower back [45]. Additionally, reduced freedom of movement and altered balance may elevate fall risk due to continuous postural adjustments [45].

While short-term benefits are evident, prolonged use of shoulder exoskeletons may lead to neuromuscular adaptations or even user dependency. Studies have raised concerns about muscle deconditioning and restricted natural movement due to structural rigidity [16,20,46].

Such effects were not evaluated in the present study but warrant consideration when interpreting increased load tolerance.

User comfort is critical for long-term adoption. Du et al. [21] evaluated 41 construction workers using the Hilti EXO-01 over two days of regular work activities. While 66% indicated willingness to use the device if provided by their employer, concerns were raised regarding comfort and usability. Participants suggested improvements such as better compatibility with harnesses and toolbelts, easier adjustment and cleaning, and a more close-fitting design to allow work in confined spaces.

In summary, shoulder exoskeletons demonstrated an ability to increase perceived unilateral overhead load tolerance during short-term duration, controlled tasks. However, these findings should not be interpreted as evidence of improved safety, reduced injury risk, or sustained performance benefits. Exoskeletons should be used selectively for specific tasks rather than continuously throughout the workday. Future research incorporating longer exposure durations and injury-related measures is needed to improve ergonomic safety and long-term effectiveness.

### 4.3 Influence of body archetypes on load capacity

Firstly, when characterizing the current sample, notable anthropometric differences were observed when compared to the reference population from the Anthropometric Survey of U.S. Army Personnel (ANSUR) database, which is based on U.S. military personnel [20]. Specifically, women in our sample were taller and had greater bust and upper arm girth, with mean values of 168 cm (SD = 6.2), 107.2 cm (SD = 13.4), and 32.4 cm (SD = 4.7), respectively. In contrast, the ANSUR female reference values were 162 cm (SD = 6.42), 94.69 cm (SD = 8.27), and 30.56 cm (SD = 3.08). Statistical testing was not conducted to determine the significance of these differences.

Conversely, males in our sample were slightly shorter and had smaller bust and upper arm girth, with mean values of 173.7 cm (SD = 7.1), 107.2 cm (SD = 13.4), and 32.9 cm (SD = 5.4), compared to the ANSUR male reference values of 175.6 cm (SD = 6.86), 105.87 cm (SD = 8.74), and 35.81 cm (SD = 3.46). These deviations highlight the distinct anthropometric profile of construction workers, likely shaped by regional, ethnic, and occupational lifestyle factors.

Given that the ANSUR database reflects a military population with different physical demands, these findings emphasize the importance of developing tailored ergonomic guidelines that reflect the specific characteristics of construction workers. Such efforts can contribute to safer, more inclusive, and more effective work environments.

Individuals with a larger body archetype were able to raise significantly heavier loads, 317 g more on average, compared to those with a smaller build. No significant differences were observed between medium and smaller build archetypes. This may be partially explained by physiological factors. Participants with larger builds likely possess greater muscle mass and joint stability, which could contribute to improved raising capacity.finding suggest that body archetype may be an important factor to consider when evaluating load tolerance during unilateral overhead tasks. Shoulder passive exoskeletons may interact differently with users of varying body archetypes, as larger body dimensions may facilitate improved alignment between the user and the device support structures. Customizable or adjustable designs are recommended to accommodate diverse body types and optimize support.

### 4.4 Practical implications

The selected drilling task was intended to represent a commonly performed unilateral overhead activity across several construction trades [21,46]. The force requirement (70N) reflects average drilling forces [27,47], while the fixed task duration and frequency were chosen to mimic high-frequency overhead work from reported in field measurements. Although real-world tasks involve variability in force and posture, the standardized task selected allowed controlled comparison of perceived load tolerance with and without exoskeleton assistance.

From a practical perspective, the estimated maximum acceptable loads derived under these conditions should be interpreted as task-specific, rather than a direct field safety threshold. The findings indicate how perceived load tolerance shifts under sustained unilateral overhead demands and how the exoskeleton alters this tolerance. This information can be used to support tool-weight selection and the identification of overhead tasks where exoskeleton assistance may be more or less beneficial.

The reported findings indicated that perceived load tolerance during unilateral overhead drilling is more strongly influenced by user characteristics and exoskeleton support than by changes in shoulder elevation within the tested range. The consistent shift in acceptable loads with exoskeleton use suggests that these devices make overhead tasks feel less demanding to the user, without changing what the task physically requires. Additionally, the differences in load tolerance noted between sex and body archetypes, highlight that unilateral overhead work cannot be characterized using a single load criterion and requires task and user specific interpretation.

### 4.5 Limitations

Despite providing valuable insights into maximal permissive loads on unilateral overhead tasks and how passive shoulder exoskeletons and body archetypes influence those outcomes; this study has several limitations. Firstly, this is a short-term study, with two interventions of three conditions lasting 5 minutes each. Therefore, it did not capture fatigue accumulation, exoskeletons adaption, or performance changes over time. Future studies should incorporate longitudinal designs to better understand the long-term effects of exoskeleton use. Furthermore, although the sample size was large enough to examine the effect of exoskeleton use on selected drill weights, it may have been inadequate to detect statistically significant effects for certain variables, particularly sex. The relatively small and potentially imbalanced sample limits the robustness of subgroup comparisons (e.g., between sexes). Therefore, future studies with larger and more balanced samples are needed to more adequately assess the effects of sex on drill weight selection. Additionally, the use of self-selected load limits was based on subjective perception, introducing variability and potential bias. This study is part of a broader research initiative that includes objective physiological measurements. Future publications will examine the relationship between perceived exertion and physiological strain, with particular attention to the influence of exoskeleton use.

## Conclusions

This study reports the estimated maximum acceptable loads for unilateral overhead lifting tasks using a Psychophysical approach, examining how passive shoulder exoskeletons and body archetypes influence these guidelines. The experimental task involved fixed frequency simulated drilling against a custom-built frame at shoulder elevations of 90°, 120°, and 150°, with participants self-selecting the drill weight. Testing was conducted over two intervention days, one with the exoskeleton and one with usual methods (i.e., control).

After controlling for several important variables, findings revealed a positive effect of the exoskeleton, with an average increase of 340 g (roughly equivalent to the weight of a standard cordless drill battery) compared to the control condition. While this suggests that participants selected slightly heavier loads when using the exoskeleton, the magnitude of this increase was relatively small, indicating that any load compensation associated with exoskeleton use may be modest in practical terms. Drill weight selection remained consistent across all shoulder elevations. Males lifted 300 g more than females, and individuals with larger body builds lifted 317 g more than those with smaller builds. These results highlight the effect of exoskeletons, the importance of considering sex and anthropometric characteristics and individual capacity in overhead work, and in exoskeleton fit and performance.

Lastly, the estimated maximum acceptable load for unilateral overhead tasks was generally below 3 kg for both control and exoskeleton conditions, revealing a mismatch between safe lifting thresholds and the heavier tools commonly used in construction. These findings suggest that exoskeletons should not be viewed as a standalone solution. Instead, their implementation should be complemented by task redesign, tool optimization, and broader ergonomic strategies to reduce excessive physical loads.

## Supporting information

S1 Data Dataset for psychophysical estimates of maximal acceptable loads during unilateral overhead work. Raw and processed data collected from 29 construction workers across exoskeleton and control intervention conditions, including drill weights selected at each shoulder elevation (90°, 120°, 150°), participant anthropometric measurements, body archetype assignments, and demographic information used for mixed linear regression analyses.
(XLSX)
